# Imaging analysis for muscle stem cells and regeneration

**DOI:** 10.3389/fcell.2024.1411401

**Published:** 2024-05-07

**Authors:** Smrithi Karthikeyan, Atsushi Asakura

**Affiliations:** ^1^ Stem Cell Institute, University of Minnesota Medical School, Minneapolis, MN, United States; ^2^ Greg Marzolf Jr. Muscular Dystrophy Center, University of Minnesota Medical School, Minneapolis, MN, United States; ^3^ Department of Neurology, University of Minnesota Medical School, Minneapolis, MN, United States

**Keywords:** myogenesis, muscle stem cell, muscle regeneration, Duchenne muscular dystrophy, satellite cell, endothelial cell, skeletal muscle, niche

## Abstract

Composed of a diverse variety of cells, the skeletal muscle is one of the body’s tissues with the remarkable ability to regenerate after injury. One of the key players in the regeneration process is the muscle satellite cell (MuSC), a stem cell population for skeletal muscle, as it is the source of new myofibers. Maintaining MuSC quiescence during homeostasis involves complex interactions between MuSCs and other cells in their corresponding niche in adult skeletal muscle. After the injury, MuSCs are activated to enter the cell cycle for cell proliferation and differentiate into myotubes, followed by mature myofibers to regenerate muscle. Despite decades of research, the exact mechanisms underlying MuSC maintenance and activation remain elusive. Traditional methods of analyzing MuSCs, including cell cultures, animal models, and gene expression analyses, provide some insight into MuSC biology but lack the ability to replicate the 3-dimensional (3-D) *in vivo* muscle environment and capture dynamic processes comprehensively. Recent advancements in imaging technology, including confocal, intra-vital, and multi-photon microscopies, provide promising avenues for dynamic MuSC morphology and behavior to be observed and characterized. This chapter aims to review 3-D and live-imaging methods that have contributed to uncovering insights into MuSC behavior, morphology changes, interactions within the muscle niche, and internal signaling pathways during the quiescence to activation (Q-A) transition. Integrating advanced imaging modalities and computational tools provides a new avenue for studying complex biological processes in skeletal muscle regeneration and muscle degenerative diseases such as sarcopenia and Duchenne muscular dystrophy (DMD).

## Introduction

As the most abundant tissue in the human body, the skeletal muscle is a highly ordered tissue that performs the following vital functions for the body, including breathing, posture maintenance, and body locomotion. The skeletal muscle tissue itself contains a variety of the following different cell types: multinucleated muscle fibers (myofibers), muscle stem cells (also known as muscle satellite cells or MuSCs), endothelial cells (ECs), pericytes (PCs), side population (SP) cells, mesenchymal progenitors/fibro-adipogenic progenitors (FAPs), motor neurons, Schwann cells, muscle spindle cells for peripheral nerves, immune cells, and undefined fibroblastic cells (Asakura et al., 2001; Asakura et al., 2002; Verma et al., 2018; Wosczyna et al., 2018; Cutler et al., 2022). The multinucleated myofibers are long-lived and elongated cells with little turnover without disease or injury (Kann et al., 2021; Krauss and Kann, 2023). However, following injury, the skeletal muscle has the unique ability to regenerate.

Skeletal muscle regeneration is a complex process orchestrated by the dynamic interplay between the diverse variety of cells in the skeletal tissue. The leading player in the regeneration process is the resident MuSCs, a stem cell population for skeletal myocytes—the source of new myofibers in normal conditions (Asakura et al., 2001). During homeostasis, MuSCs maintain a quiescent state in which the cells have reversibly exited the cell cycle but retain their ability to re-enter the division process in response to specific environmental cues. MuSC quiescence is a state that must be actively maintained by a combination of cell-autonomous factors and external signals provided by the MuSC niche (Kann et al., 2021; Ma and Mourkioti, 2023). Upon injury, MuSCs undergo an activation process in which they activate, proliferate, and differentiate to form new muscle fibers. In addition, MuSCs undergo a self-renewal process to replenish the resident stem cell pool, allowing the muscle to undergo the regeneration process for multiple injuries (Kann et al., 2021; Krauss and Kann, 2023). Although several decades of research have gone into understanding the signaling factors and cellular interactions involved in the MuSC quiescence-to-activation transition (Q-A transition), the overall mechanism of this process still requires further exploration. Understanding the mechanisms of the Q-A transition is vital for understanding/modeling the skeletal muscle regeneration process in general.

Duchenne muscular dystrophy (DMD) is a progressive neuromuscular disease caused by *dystrophin* deficiency that inevitably leads to death. Although well characterized concerning muscle lesions in DMD, it is still not fully understood why MuSC-induced muscle regeneration fades with progression, ultimately resulting in skeletal muscle atrophy. Furthermore, the possible vascular changes that may affect the mechanism of skeletal muscle regeneration are poorly understood (Latroche et al., 2015a).

Historically, *in vivo* MuSC behavior has been inferred through histological analysis of skeletal muscle tissue cross-sections, cell cultures of single myofibers, and *ex vivo* studies utilizing isolated MuSCs (Asakura et al., 2001; Asakura et al., 2002). Although these approaches have provided great insight into MuSC behavior, they cannot replicate the true *in vivo* environment in which the MuSCs receive several signaling cues from their niche. In addition, these methods only provide a snapshot view, which does not represent the entirety of MuSC dynamics (Ratnayake and Currie, 2017; Ma et al., 2022).

**FIGURE 1:**
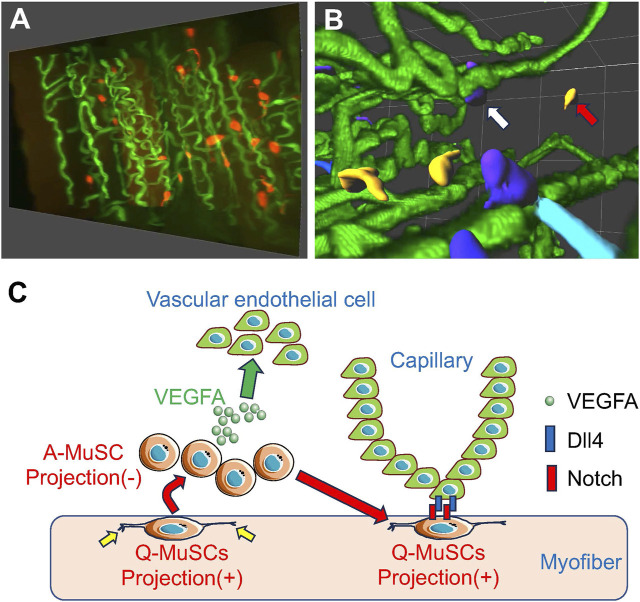
MuSCs reside in close proximity to capillary endothelial cells for MuSC self-renewal **(A)** Snapshots of MuSCs (red) and capillaries (green) from a 3-D image of mouse skeletal muscle. **(B)** Images of MuSCs are colored based on their distance from the nearest capillary. Capillaries (green) from a 3-D image of mouse skeletal muscle. Capillaries (green). Orange cells indicate activated MuSCs away from the capillaries (red arrows), and dark blue cells indicate quiescent MuSCs close to the capillaries (white arrows). **(C)** When activated (A)-MuSCs express VEGFA during regeneration, vascular endothelial cells are recruited in the vicinity of the MuSCs, forming a vascular niche. Subsequently, Notch signaling in MuSCs is activated by Dll4 derived from adjacent vascular endothelial cells, inducing self-renewal of Q-MuSCs. Q-MuSCs have longer, multiple projections (yellow allows), which regress after activation.

With the recent advancements in both *in vivo* models and microscopy technologies, scientists have overcome some of the above issues in previous research methods. The combination of advanced imaging modalities (such as confocal, multi-photon, and spinning disk microscopes), improved stability of fluorescent proteins, and expanded computational power for image analysis has given researchers a whole new set of tools to study complex biological processes in detail, including, MuSC self-renewal, proliferation and activation during muscle regeneration and DMD progression (Figure 1; Verma et al., 2016; Ratnayake and Currie, 2017; Verma et al., 2018; Kann et al., 2022; Ma et al., 2022). This chapter will review several imaging methods for MuSC behavior in the skeletal muscle. With these imaging methods, we intend to review several advances in understanding the mechanisms behind the MuSC Q-A transition, including changes in MuSC morphology, interactions with other cells in the MuSC niche, and internal signaling pathways.

## Previous work and barriers to studying the Q-A transition and MuSC morphology

Historically, MuSCs have been described as small, fusiform cells with a high nuclear-to-cytoplasmic ratio (Kann et al., 2021; Krauss and Kann, 2023) (Table 1). With improvements to imaging and muscle preparation techniques, studies have revealed that quiescent MuSCs are a heterogeneous population with cellular projections. In a study conducted by Schmalbruch in 1978, rat hind-limb skeletal muscles were freeze-fractured, and an electron microscope was used to characterize MuSC shape and its relationship to its underlying muscle fiber. After fracture examination, this study noted that most MuSCs had projections that indented the surface of the underlying muscle fiber. In addition, several cross-sections showcased how the underlying muscle fibers had projections that embraced the MuSCs (Schmalbruch, 1978). An example of such an interaction is the co-localization of MuSCs and vascular ECs. It has been suggested that MuSCs are preferentially located near blood vessels (Christov et al., 2007). However, this interaction may be underestimated in a typical two-dimensional (2-D) analysis. This scenario underscores the importance of analyzing cell-cell interactions in muscle tissue in their native three-dimensional (3-D) state. These observations lead to the hypotheses that 1) there is a cellular cross-talk between MuSCs and the surrounding niche cells, such as myofibers and ECs, and 2) the MuSC projections play a role in their renowned migratory movements.

**TABLE 1:** Methods for *in vivo* and *ex vivo* MuSC characterization (A) Freeze Fracture Electron Microscopy is a technique of physically breaking apart (fracturing) a frozen biological sample followed by electron microscopic observation. This method analyzes cells, typically looking at the cell membrane and lipid-containing structure. (B) Myofiber/MuSC Culture is an *ex vivo* study in which MuSCs or myofibers are isolated and cultured. Behavior is then characterized via histological analysis. (C) Confocal or light-sheet fluorescence microscopy is a technique that allows for the 3-D optical resolution of tissues. Used in conjunction with fluorescence, it allows for the labeling of cells via cell-specific proteins. (D) Intravital Microscopy is a live imaging technique that allows visualization of cells *in vivo*.

Imaging Method	Description	Pros	Cons	Citations
(A) Freeze Fracture Electron Microscopy	Method for the cross-sectional analysis of cells (typically looking at the cell membrane and lipid-containing structures)	Preservation of native structures and excellent resolution for membrane structures	Artifact introduction and 2D analysis	Schmalbruch (1978)
(B) Myofiber/MuSC Culture	A technique allowing 3-D optical resolution of tissues. Used in conjunction with immunofluorescence, it allows for labeling cells via cell-specific proteins	Facilitates experiments in understanding mechanistic pathways	Not always physiologically relevant, not permanently preserving the native structure	Asakura et al. (2001), Asakura et al. (2002), Many other papers are available
(C) 3D analysis by Confocal or Light- Sheet Fluorescence Microscopy	Technique allowing 3-D optical resolution of tissues. Used in conjunction with immunofluorescence allows for the labeling of cells via cell-specific proteins	Improved optical sectioning and high-resolution imaging allow for more quantitative analysis methods via multi-channel imaging	Cost, slower imaging speeds, and limited penetration depth	Verma et al. (2016), Verma et al. (2018), Kann et al. (2022), Ma et al. (2022), Karthikeyan et al. (2023a), Karthikeyan et al. (2023b), Ma et al. (2023)
(D) Intravital Imaging	Live imaging technique allows visualization of tissues *in vivo* or *ex vivo*	Real-time observation and physiological relevance	Limited penetration depth, photobleaching, and limited field of view	Siegel et al. (2011), Webster et al. (2016a), Webster et al. (2016b), Ratnayake and Currie (2017), Konagaya et al. (2020), Evano et al. (2023), Jacobsen et al. (2023)

A limitation of the above study is that it is a static analysis. While it provided valuable insights into MuSC behavior, such static analyses from cross-sections only provide a snapshot of MuSC behavior. As a result, MuSC dynamic behavior (both in quiescence and during regeneration) is left to be inferred (Christov et al., 2007; Ratnayake and Currie, 2017). This limitation highlights the need for more advanced techniques that can capture the dynamic nature of MuSCs, especially during the Q-A transition after injury.

In general, studying MuSC quiescence has proven difficult as the isolation of MuSCs leads to rapid activation. Such sensitivity to niche perturbations prevents early examination of cellular events in the Q-A transition. Today, the identification of quiescence-promoting factors has mainly come from loss-of-function experiments in which the loss of such factors led to an activated MuSC phenotype. The mechanisms by which these factors maintain MuSC quiescence and how their dynamics change during the Q-A transition have remained unclear until recently with the advent of imaging advancements (Kann et al., 2021; Krauss and Kann, 2023). These recent advancements in imaging techniques have opened up new possibilities for studying MuSCs, instilling optimism in the field and stimulating further research into the field.

## A closer look into MuSC projections

### Fluorescent intravital imaging of MuSCs

One prominent imaging advancement is the visualization of live tissues of living organisms utilizing intravital microscopy. Such microscopy allows scientists to visualize MuSC dynamics in muscle *in vivo* in model organisms like mice and zebrafish. In a review of various MuSC live imaging studies, Siegel et al. (2011) demonstrated that time-lapse imaging studies of mouse myofiber-associated MuSCs have provided new data on the dynamic migration on the myofibers, and timing and direction of MuSC division following single myofiber cultures, revealing persistent differences in the behavior of daughter cells in planar and vertical divisions. Webster et al. (2016a) established a stepwise protocol to measure the behavior of fluorescently labeled MuSCs during homeostasis quantitatively and after mouse muscle injury. Using 3-D time-lapse intravital imaging to directly visualize the regenerating mouse tibialis anterior (TA) muscle in living mice, they showed that extracellular matrix remnants of injured skeletal muscle fibers dominate the behavior of MuSCs during regeneration as ghost fibers. After the injury, MuSC migration and cell division were primarily oriented bidirectionally along the longitudinal axis of the ghost fiber, allowing MuSCs to spread throughout the ghost fiber. Thus, ghost fibers are autonomous structural units required for proportional regeneration after tissue injury (Webster et al., 2016b). Evano et al. (2023) demonstrated *in vivo* intravital imaging for MuSC-mediated muscle regeneration using intact flexor digitorum brevis (FDB) muscle in mice. After several hours of *in vivo* imaging of regenerating muscle to monitor the initial response to muscle injury, cell migration, cell division, and cell fusion of MuSCs using two-photon microscopy or standard confocal microscopy. Konagaya et al. (2020) utilized an intravital imaging system for measuring migration rate, extracellular signal-regulated kinase (ERK) activity, and cell cycle of MuSCs within mouse TA muscle. Jacobsen et al. injected adult mice with fluorescent dextran and performed intravital imaging to assess *in vivo* microvascular perfusion of the gluteus maximus (GM) muscle. They concluded that angiogenesis precedes myogenesis in regeneration after muscle injury (Jacobsen et al., 2023). Collins et al. (2024) utilized whole-mount imaging in 3-D with fluorescently labeled regenerating muscle and demonstrated that muscle fibers form via two distinct phases: Cell fusion of MuSCs first establishes muscle fibers, then MuSC-muscle fiber fusion enlarges the fibers. They also found the essential roles of residual muscle fiber basement membrane that promotes myogenic fusion and orients regenerating muscle fibers.

Ratnayake and Currie highlight how this live-imaging technique has provided insights into MuSC morphology during the zebrafish regeneration. The directional migration behavior of MuSCs after injury was observed using live-microscopic imaging and fluorescently labeled zebrafish larvae. Utilizing a double transgenic zebrafish line in which differentiated myofibers were labeled via mCherry and MuSCs were labeled with GFP (Myf5 expressing cells), laser ablation was used to create a minor focal injury by ablating a small number of myofibers. Then, the zebrafish larvae were allowed to resolve this injury while being time-lapsed imaged for 48 h. The images from this experiment revealed that the skeletal muscle regeneration process is marked by stages with distinct MuSC morphologies/behaviors. MuSCs migrating to the wound site displayed a polarized bipolar phenotype with extended cytoplasmic projections. At the wound site, the initially bipolar MuSCs change to a more rounded shape to associate with the dying myofibers to generate a progenitor pool. The subsequent progenitors disassociate from the dying myofiber and assume a bipolar phenotype. The progenitors were noted to interact with each other via their cytoplasmic extensions. Finally, the uninjured myofibers guide these progenitor cells to regions of injury in which the progenitors differentiate into myofibers (Ratnayake and Currie, 2017).

### Investigating the role of filopodia in myoblast fusion using time-lapse confocal imaging

Focusing on the last stages of the myofiber regeneration process, an imaging study conducted by Hammers et al. (2021) elucidates the role of cellular projections in myoblast fusion. Myoblast fusion is generally described as merging two opposing myoblast lipid bilayers. This process involves several widely expressed protein classes: cytoskeleton elements, phagocytosis receptors, and calcium-sensing membrane repair proteins. In addition, thin, actin-filled projections have been observed to be involved in the fusion process using confocal time-lapse imaging (Hammers et al., 2021).

To determine if these projections are filopodia, the class x myosin (Myo10) expression pattern was monitored via immunofluorescence in cultures of differentiating MuSCs. Myo10 presence is a hallmark of filopodia as it is a molecular motor associated with the initiation/elongation of filopodia and transport within filopodia. Immunofluorescent confocal images of the differentiated myoblast cultures showcased that the projections were Myo10-positive and, therefore, were filopodia. In subsequent experiments, Myo10-knockout MuSCs did not form filopodia and continued with cell fusion, suggesting that Myo10 (and, therefore, filopodia) is essential for myoblast fusion (Hammers et al., 2021). In exploring filopodia’s role in the fusion process, further fluorescence imaging experiments showcased how muscle fusion proteins Myomaker and Myomixer localize on myoblast filopodia (Hammers et al., 2021). The results of this study illustrate the great potential of confocal imaging with the level of detail this imaging advancement provides in studying protein expression in the muscle regeneration process.

### Investigation of intracellular signaling and MuSC projections

Interestingly, fluorescent-labeled observation of MuSCs revealed that during quiescence, MuSCs have projections of various lengths and numbers in mice (Figure 1; Verma et al., 2018; Kann et al., 2022; Ma et al., 2022). The morphology of the projections is consistent with the dynamic and motile structure of MuSCs, which form actin-based filopodia at their distal ends. Filopodia are essential for signaling ligand sensing, cell-cell interactions, and cell migration. These MuSC projections are very long, often branched, and highly heterogeneous, and loss of these projections is observed in activated MuSCs, which require active motility (Kann et al., 2022; Ma et al., 2022). As demonstrated in the previous studies above, MuSCs change their shapes and structure during different stages of the regeneration process. Such changes in cell shapes and structure have been reported to be driven by the Rho family of GTPases (Rho, Rac, and Cdc42). These membrane-bound GTPases mediate cytoskeletal rearrangements, downstream signaling, and transcriptional changes in response to other extracellular signaling cues (Burridge and Wennerberg, 2004). Previous literature denotes how an inverse relationship exists between Rac and Rho, therefore functioning as a molecular switch coordinating changes in cell morphology (Guilluy et al., 2011).

To reveal the molecular mechanisms in the Q-A transition of MuSCs with the projections via the Rho/Rac switch, tissue clearing with modified *ex vivo* single myofiber experiments was conducted in a study by Kann et al. (2022). The results of this study showcased how the long cytoplasmic projections seen in quiescent MuSCs are associated with high levels of Rac/Cdc42 activity. Therefore, Rac activity functions to promote projection outgrowth. In early activation (physiologically in response to injury), Rac activity in MuSCs is downregulated, and Rho activity increases. With increased Rho activity, MuSC projections were shown to retract, marking the beginning of the Q-A transition (Kann et al., 2022). The results of this study illustrate the great potential of tissue-clearing and confocal imaging with the level of detail this imaging advancement provides in studying intracellular signaling in quiescent MuSCs.

Ma et al. (2022) demonstrated that the transition between these different MuSC states is regulated by Piezo1, a sensing protein that promotes regeneration. While pharmacological activation of Piezo1 was shown to activate MuSCs during muscle regeneration, deletion of Piezo1 in MuSCs shifts MuSCs to less activated cells, mimicking the disease phenotype seen in DMD muscle. They also found that reactivation of Piezo1 ameliorates the morphological and regenerative defects of MuSCs in DMD muscle. These results advance our understanding of how MuSCs respond to muscle injury and demonstrate that the sensing protein Piezo1 is essential for MuSC activation in muscle regeneration.

### Investigation of MuSC quiescence maintenance via cellular cross-talk with surrounding vasculature niche

One of the critical components of the skeletal muscle niche is the vascular network that stretches within skeletal muscle and envelopes MuSCs and muscle fibers. ECs, which constitute the vascular network, have been reported to exist in close proximity to MuSCs, and the number of MuSCs significantly correlates with the density of capillaries. Myofibers promote angiogenesis via the expression of vascular endothelial growth factor (VEGF) (Latroche et al., 2015b). However, it has yet to be verified whether ECs in the vicinity of MuSCs are involved in MuSC activation, self-renewal, and maintenance of quiescence.

Using our proprietary skeletal muscle-specific tissue-clearing technology, mice in which MuSCs and ECs are simultaneously fluorescently labeled in skeletal muscle, and confocal microscopy, we have successfully visualized the spatial relationship between the complex network of capillaries and MuSCs in 3-D (Verma et al., 2016; Karthikeyan et al., 2023a; Karthikeyan et al., 2023b). This analysis revealed that in uninjured skeletal muscle, 40%–80% of MuSCs reside in the vicinity of ECs, indicating that MuSCs residing in ECs have more of a stem cell character than those living farther away. Furthermore, transcriptome analysis showed that quiescent and activated MuSCs express high levels of VEGFA, a subtype of VEGF, and VEGFA gene deletion experiments in MuSCs indicated that MuSC-derived VEGFA is essential for recruiting ECs expressing the VEGFA receptor (Figure 1; Verma et al., 2018).

To further explore how adjacent ECs regulate MuSC self-renewal and quiescence, transcriptome analysis identified Delta-Like-4 (Dll4), one of the ligands for Notch, as being strongly expressed in ECs. Previous studies have shown that activation of the Notch pathway is essential for MuSC self-renewal and quiescence maintenance, and our experiments with co-cultures of MuSC and ECs revealed that Notch signaling in MuSC is activated by Dll4, a Notch ligand, derived from ECs, potentially via direct cell-cell contact. These results indicate that MuSCs recruit ECs via VEGFA to form vascular niches and that Notch signaling-mediated cross-talk between ECs and MuSCs is essential for MuSC replenishment and maintenance (Figure 1; Verma et al., 2018).

Our recent studies have shown that ECs secrete the Notch ligand Dll4 as an extracellular domain and activate Notch2 expressed in muscle fibers. Dll4 with this extracellular domain induces disuse muscle atrophy and diabetes-induced muscle atrophy via Notch2. These results demonstrate that direct cellular contact is not essential for the activation of Notch signaling and prove a new mechanism by which Notch ligands secreted from surrounding cells can activate Notch signaling. Furthermore, this activation of Notch2 by ECs-derived Dll4 is essential for the progression of muscle atrophy seen in these pathologies, suggesting that this secreted Dll4 may be a potential therapeutic target for disuse muscle and diabetes-induced muscle atrophy (Fujimaki et al., 2022). Eliazer demonstrated that Dll4 is expressed in myofibers, and myofiber-derived Dll4 is essential for maintaining MuSCs (Eliazer et al., 2020).

### Macrophages as a transient muscle stem cell niche

Macrophages (MPs) have long been known to be essential actors of skeletal muscle regeneration, and the lack of an MP subset severely impairs muscle regeneration (Manole et al., 2021; Scala et al., 2021; Henrot et al., 2023). In the early phase of skeletal muscle regeneration, the innate immune response plays essential roles in the activation of neutrophils, mast cells, and the complement system, resulting in the recruitment of monocytes at the damaged muscle region. The monocytes can differentiate into two different MP phenotypes by different acting stimuli. The migrated monocytes give rise to matured MPs with first a pro-inflammatory phenotype (M1-MPs) within 24 h after muscle damage, followed by an anti-inflammatory phenotype (M2-MPs) within 2–4 days. Both MPs co-operatory regulate MuSCs to promote muscle regeneration: Initially, M1-MPs promote clearance of necrotic debris and suppress MuSC differentiation, followed by the polarization shift from M1 to M2- MPs that promote muscle regeneration by attenuating inflammation and stimulating MuSC proliferation, migration, and differentiation. M2-MPs also play a role in the angiogenesis-myogenesis coupling by promoting capillaries and myotube formation (Latroche et al., 2017).

Recent live imaging studies using zebrafish have unveiled a novel regulatory mechanism of MuSCs by MPs (Ratnayake et al., 2021). These studies have identified a specific subset of MPs termed “dwelling MPs” within the injury site. These dwelling MPs provide a transient niche for MuSC proliferation through the secretion of specific molecules, such as the cytokine nicotinamide phosphoribosyltransferase (NAMPT), which acts through the C-C motif chemokine receptor type 5 (Ccr5) expressed on MuSCs. The live imaging analysis has demonstrated that these specific MPs can directly interact with MuSCs, supplying proliferation-inducing cues for muscle regeneration. These findings open up new avenues for research and deepen our understanding of the complex interactions in muscle regeneration.

In summary, analyzing skeletal muscles that maintain three-dimensional structures will be essential to better understanding MuSCs and the niche cells surrounding them, a dynamic understanding of aging-induced muscle weakness and skeletal muscle diseases, such as DMD, and the development of therapeutic strategies.

## Figures and Tables

**Table:** 

**(DMD)**	Duchenne muscular dystrophy
**(MuSC)**	Muscle satellite cell
**(EC)**	Endothelial cell
**(PC)**	Pericytes
**(SP)**	Side population
**(FAP)**	Fibro-adipogenic progenitors
**(2-D)**	Two-dimensional
**3-D)**	Three-dimensional
**(Q-A)**	Quiescence to activation
**(GM)**	Gluteus maximus
**(TA)**	Tibialis anterior
**(Dll4)**	Delta-Like-4
**(VEGF)**	Vascular endothelial growth factor
